# Neuregulin-1β Partially Improves Cardiac Function in Volume-Overload Heart Failure Through Regulation of Abnormal Calcium Handling

**DOI:** 10.3389/fphar.2019.00616

**Published:** 2019-06-21

**Authors:** Xuehui Wang, Xiaozhen Zhuo, Jie Gao, Huibing Liu, Fei Lin, Aiqun Ma

**Affiliations:** ^1^Department of Cardiovascular Medicine, First Affiliated Hospital of Xinxiang Medical University, Weihui, China; ^2^Department of Cardiovascular Medicine, First Affiliated Hospital of Xi’an Jiaotong University, Xi’an, China

**Keywords:** heart failure, neuregulin-1β, L-type calcium channel, calcium handling, SERCA2a

## Abstract

**Background:** Neuregulin (NRG-1), an essential stress-mediated paracrine growth factor, has a cardioprotective effect in failing heart. However, the underlying mechanism remains unclear. The role of NRG-1β in heart failure (HF) rats was examined.

**Methods and Results:** Volume-overload HF rat model was created by aortocaval fistula surgery. The sham-operated (SO) rats received the same surgical intervention without the fistula. Thirty-five HF rats were injected with NRG-1β (NRG, 10 μg/kg·d) *via* the tail vein for 7 days, whereas 35 HF rats and 20 SO rats were injected with the same dose of saline. The echocardiographic findings showed left ventricular dilatation, systolic and diastolic dysfunction, and QTc interval prolongation in HF rats. The NRG-1β treatment attenuated the ventricular remodeling and shortened the QTc interval. Patch clamp recordings showed I_Ca-L_ was significantly decreased in the HF group, and NRG-1β treatment attenuated the decreased I_Ca-L_. No significant differences in the kinetic properties of I_Ca-L_ were observed. The expressions of Cav1.2 and SERCA2a were significantly reduced, but the expression level of NCX1 was increased dramatically in the HF group. NRG-1β treatment could partially prevent the decrease of Cav1.2 and SERCA2a, and the increase of NCX1 in HF rats.

**Conclusions:** NRG-1β could partly attenuate the heart function deterioration in the volume-overload model. Reduced function and expression of calcium transportation-related proteins might be the underlying mechanism.

## Introduction

Heart failure (HF) is a devastating condition with limited treatment options, and it has been a challenge for clinicians now and in the future (Braunwald, 2015; Mitter and Yancy, 2017). Although the pharmacologic therapies and device had made advance in HF treatments, the survival rate in patients with advanced congestive heart failure (CHF) is limited. Thus, novel treatment strategies and potential therapeutic targets are urgently required. Neuregulin (NRG-1), an important stress-mediated paracrine growth factor, has been shown a cardioprotective effect in failing heart (Galindo et al., 2014; Shakeri et al., 2018). However, the underlying mechanism remains unclear.

Abnormal Ca^2+^ handling in cardiomyocytes has been increasingly implicated in the development of HF and has been shown to influence excitation-contraction coupling (ECC) and cell function, which leads to systolic dysfunction and arrhythmias (Johnson and Antoons, 2018). Recent studies have revealed that intracellular Ca^2+^ dynamics are impaired before myocardial contractility is weakened. As a result, pharmacologic modification of cellular Ca^2+^ handling has become an alternative treatment and prevention strategy for HF. The proteins involved in Ca^2+^ handling, including the L-type voltage-dependent Ca^2+^ channel alpha 1C subunit (Cav1.2), SR Ca^2+^ ATPase (SERCA2a), and sodium-calcium exchanger (NCX), are essential for Ca^2+^ homeostasis and represent potential targets for HF therapy (Hasenfuss and Pieske, 2002; Gorski et al., 2015).

Neuregulin-1β (NRG-1β) belongs to a family of growth factors released by microvascular endothelial cells in the heart and other organs (Odiete et al., 2012; Galindo et al., 2014; Wang et al., 2018). NRG-1β along with its receptors (ErbB2-4) is required for cardiac development and heart function (Odiete et al., 2012; Galindo et al., 2014). In the adult, cardiac-specific deletion in NRG-1, ErbB4, or ErbB2 results in ventricular dilatation, dysfunction, and increased susceptibility to anthracycline exposure (Ozcelik et al., 2002; Liu et al., 2005). Conversely, enhancement of NRG-1 signaling preserves cardiac function and survival in chronic HF models through the protection of contractile proteins (Kuramochi et al., 2006; Gu et al., 2010), regulation of energy utilization, promotion of cell survival, and cell division (Zhao et al., 1998; Fukazawa et al., 2003). Because NRG-1β is considered as a potential therapeutic agent for HF treatment, it is important to identify the mechanism of its action. Studies have revealed that NRG-1β alters Ca^2+^ handling and increases intracellular Ca^2+^ concentration in hippocampal neurons (Schapansky et al., 2009). Also, NRG-1β could regulate Ca^2+^ homeostasis in cardiac myocytes by enhancing the activity and expression of SERCA2 proteins. However, it is unclear whether the therapeutic effect of NRG-1β on HF is mediated by regulating Ca^2+^ homeostasis. The present study investigated the effects and underlying mechanism of NRG-1β on volume-overload HF rats.

## Methods

### Materials

Male Sprague–Dawley rats (160–180 g) were obtained from the Experimental Animal Center of Xi’an Jiaotong University (Shaanxi, China). Experimental procedures were approved by the Care of Experimental Animals Committee of First Affiliated Hospital of Xi’an Jiaotong University according to U.S. Animal Welfare Act, and all animals were treated humanely and with regard for alleviation of pain in accordance with the Guide for the Care and Use of Laboratory Animals (Institute of Laboratory Animal Resources, National Research Council 2011). Chemicals and antibodies used were as follows: NRG-1β from ProSpec-Tany TechnoGene (Nes Ziona, Israel), rat brain natriuretic peptide (BNP-45) enzyme-linked immunosorbent assay (ELISA) kit from Assaypro (MO, USA), enhanced chemiluminescence (ECL) from Pierce (IL, USA), type II collagenase from Worthington (NJ, USA), Cav1.2 antibody from Alomone (Jerusalem, Israel), NCX1 antibody from Millipore (MA, USA), SERCA2 antibody, β-actin antibody, and horseradish peroxidase (HRP)-labeled secondary antibody from Santa Cruz Biotechnology (CA, USA).

### Animal Model Preparation and NRG-1β Treatment

**Figure 1:**
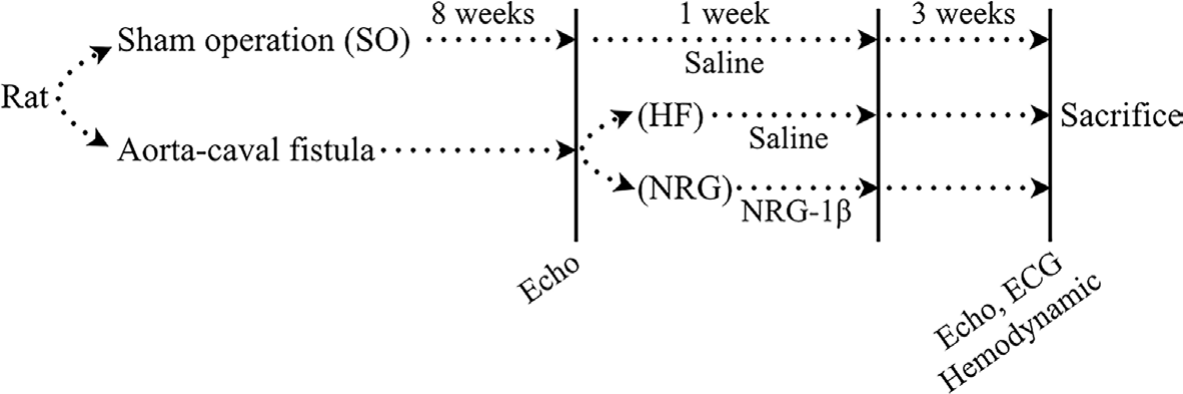
Schematic depiction of the animal study. Volume-overload heart failure (HF) was established in rats by aorta-caval fistula surgery. Sham-operated rats (SO group) received the same operation without the fistula. HF rats were confirmed at 8 weeks after surgery by echocardiographic measurement. HF rats were injected with NRG-1β (10 μg/kg·d) by intravenous injection *via* the tail vein for 7 days as previously reported. The rats in the HF group and the SO group were administered with the same dose of saline. All of the rats were maintained for 12 weeks. Echocardiographic and hemodynamic measurements measured cardiac function. Rats were sacrificed after the last measurement

The animal study design was illustrated in **Figure 1**. HF model was established in rats as previously described (Du et al., 2007). Briefly, the 82 rats were anesthetized with pentobarbital sodium (30 mg/kg, i.p.) (Yang et al., 2011) and volume-overload HF (HF group) was established by aorta-caval fistula (ACF) surgery (Flaim et al., 1979). The sham-operated rats (SO group) received the same surgery without the fistula. Briefly, after anesthesia, the abdomen was opened layer by layer, and then the inferior vena cava and the abdominal aorta was exposed by gently putting the intestinal system aside. A “U” type suture was created on the inferior vena cava. And, then, the shared wall between inferior vena cava and the abdominal aorta was grasped through a longitudinal incision made in the inferior vena cava, and a fistula was created between the two vessels (side to side, 1.0–1.2 mm in length) with an 18G peripheral venous catheter (BD, NJ, USA). The opening in the vena cava was then closed by fastening the “U” type suture. Right after the surgery, the antibiotic penicillin, 30,000 U/kg/day, was used for first 3 days.

There were 12 rats that died during and right after the surgery. Seventy HF was confirmed at 8 weeks after surgery by echocardiographic measurement. HF rats (NRG group, n = 35) were injected with NRG-1β (10 μg/kg·d) by intravenous injection *via* the tail vein for 7 days as previously reported (Liu et al., 2006; Guo et al., 2012). The rats in the HF group (n = 35) and SO group (n = 20) were administered with the same dose of saline. All of the rats were maintained for 12 weeks.

### Cardiac Function Evaluation

Echocardiographic and hemodynamic measurements measured cardiac function. For echocardiographic measurement, the rats were anesthetized by intraperitoneal injection of 10% chloral hydrate (300 mg/kg), (Lindholm et al., 2017) and the cardiac function was evaluated using the Philips IE33 ultrasound system with an ultra-band transducer of 7.5 MHz (HP, USA).

For hemodynamic measurement, a BL-420F data acquisition and analysis system (Chengdu TME Technology Co., Ltd., China) was used. A catheter (PE-50 tubing) connected to the pressure sensor was inserted into the right carotid artery of rats under sodium pentobarbital (30 mg/kg, i.p.) for measurement of intraventricular pressure curve. The QT interval was evaluated using a standard lead II ECG, and the serum level of BNP at the week of 12 was measured using an ELISA kit. Rats were sacrificed under anesthetization with sodium pentobarbital (45 mg/kg, i.p.) after the last measurement.

### Electrophysiological Recording of Single Cardiomyocytes

Left ventricular myocytes were isolated as previously described (Xi et al., 2009). Briefly, the rats were anesthetized with sodium pentobarbital (45 mg/kg, i.p.) and anti-coagulated with heparin sodium (400 IU/kg, i.p.). The heart was cannulated and digested using 300 IU/ml collagenase type II, 0.03% protease with 0.1% bovine serum albumin dissolved in Tyrode’s solution containing 0.06 mmol/L Ca^2+^ *via* a Langendorff system at 37°C. The isolated cardiomyocytes were stored in KB solution at room temperature (22–24°C). Only quiescent, rod-shaped cells showing distinct striations were selected for the experiment. L-type Ca^2+^ currents (I_Ca-L_) were recorded by whole-cell patch clamp recordings (Axopatch 700B Amplifier, Axon Instruments, USA) at room temperature (22–24°C) installed on a microscope (IX71, Olympus, Japan). The resistance of the glass electrode was 2–4 MΩ when filled with the pipette solution. The currents were filtered by a low-pass Bessel filter at 1 kHz and digitized at a sampling rate of 10 kHz (Digidata 1440A, Axon Instruments, USA). The bath solution contained (mM): choline chloride 120, CsCl 4, CaCl_2_ 1.8, MgCl_2_ 2, 4-(2-hydroxyethyl)-1-piperazineethanesulfonic acid (HEPES) 10, and glucose 10 (pH 7.40 by CsOH). The pipette solution contained (mM): CsCl 120, MgCl_2_ 2, HEPES 10, ethylene glycol tetraacetic acid (EGTA) 10, K_2_ATP 5, and tetraethylammonium ions (TEA) 10 (pH 7.40 by CsOH). KB solution contained (mM): KCl 25, KH_2_PO_4_ 10, MgCl_2_ 3, taurine 20, L-glutamic 70, EGTA 0.5, HEPES 10, and glucose 10 (pH 7.40 by KOH). The junction potential between the pipette solution and bath solution was 5 mV. All voltage applied was corrected afterward. NRG1β perfusion solution (every fresh preparation): NRG1β 50 µg was dissolved in 0.5 ml deionized water and diluted into solution with 1 µg/ml final concentration by the bath solution.

In all of the experiments, the holding potential was set at −40 mV. To obtain current–voltage (I–V) curves and steady-state activation curves, I_Ca-L_ was elicited by a single pulse of 300 ms to +70 mV from the holding potential in 5-mV increments at 0.1 Hz. The current amplitude was normalized to the cell capacitance (current density, pA/pF). Steady-state inactivation was determined with a double-pulse protocol consisting of 1,000 ms pre-pulses to voltages between −70 and +30 mV (5-mV increments), followed by a constant test pulse of 150 ms to 0 mV. The time dependence of I_Ca-L_ recovery from inactivation was studied by a double-pulse protocol that included two depolarizing pulses to 0 mV with varying interpulse intervals (10–12,800 ms) applied from the holding potential (−40 mV). The extent of recovery at each interpulse interval was obtained by expressing the amplitude of I_Ca-L_ in response to the test pulse as a fraction of the Ca^2+^ current amplitude elicited by the conditioning pulse. Data acquisition and analyses were performed using Clampfit software (Axon Instruments. USA).

### Immunofluorescence Staining

Single cardiomyocytes were isolated from the LV of the adult rat and fixed with 4% paraformaldehyde in phosphate buffered saline (PBS) at 4°C for 15 min. The cells were then incubated in 0.1% Triton-X for 15 min to permeabilize the membranes and then blocked with 10% goat serum for 10–15 min. To visualize proteins, the cells were exposed to primary antibodies overnight at 4°C and then secondary antibodies for 30 min at 37°C. The nuclei were stained with 4’,6-diamidino-2-phenylindole (DAPI; Southern Biotech, USA). Anti-Cav1.2, anti-SERCA2a, and anti-NCX1 antibody were purchased from Alomone (Jerusalem, ACC-003, Israel), Millipore (MA, AB3516P, USA), and Santa Cruz Biotechnology (CA, SC-8094, USA), and secondary antibodies, anti-rabbit488 conjugated antibody was purchased from Santa Cruz Biotechnology (CA, sc-362262, USA). Cardiomyocyte images were captured using a confocal laser-scanning microscope (Bio-Rad MRC-1024 Confocal Microscope System, USA).

### Western Blot Analysis

Membrane proteins were extracted from left ventricular myocardium tissue by centrifugation in a lysis buffer (RIPA buffer) with a protease inhibitor cocktail. The protein samples were run on sodium dodecyl sulfate-polyacrylamide gel electrophoresis gels and electrophoretically transferred to nitrocellulose membranes. The transferred proteins were incubated with a blocking solution containing 5% (w/v) dry skim milk and Tris-buffered saline with Tween-20 (TBST) for 1 h. After blocking, the membrane was treated overnight at 4°C with the primary antibodies same as immunofluorescence. After washing with TBST three times, the membrane was incubated with the HRP-labeled secondary antibody for 1 h at room temperature. The membranes were washed with TBST and visualized using ECL on X-ray film. The band intensities on the nitrocellulose membranes were quantified by densitometry scanning. The protein amount was semi-quantified by normalizing the target protein band intensities to that of β-actin.

### Statistical Analysis

All of the values are expressed as mean ± SEM. An independent sample t-test analyzed the differences between the two groups. Comparisons between three groups were tested by one-way analysis of variance (ANOVA), and the pairwise comparisons were performed with a least square difference (LSD) test. A value of *P* < 0.05 was considered significant.

## Results

### NRG-1β Improves the Cardiac Function of the Rat With Heart Failure

At week 12, heart function was evaluated (randomly selected): 42 with echocardiography (**Supplemental Figure I A**) and some of the rats were used in other experiments such us molecular biologic and electrophysiological experiments. As shown in **Table 1**, the model of volume-overload HF rats with significant LV hypertrophy and systolic dysfunction (compared with that of the rats in SO group) was successfully produced at 8 weeks after the operation based on echocardiographic data. Both the left ventricular end-systolic dimension (LVESD) and left ventricular end-diastolic dimension (LVEDD) were significantly increased, and left ejection fraction (LVEF) and fraction shortening (LVFS) were markedly reduced. Also, QTc intervals were considerably prolonged, and plasma BNP levels were raised in the HF rats (*P* < 0.05, **Table 2**, **Supplemental Figure I B**). As illustrated in **Figure 1**, the cardiac function of rats was further evaluated using echocardiographic and hemodynamic analysis at 12 weeks after surgery. NRG-1β treatment moderately reverses the changes in LVEDD, LVESD, LVEF, and LVFS in rats with HF (all *P* < 0.05, **Table 2**). Hemodynamic data also supported these findings, after NRG-1β treatment, downregulated LVESP and ± dP/dt_max_ and upregulated LVEDP in HF rats were partially reversed (all *P* < 0.01, **Table 2**).

**Table 1:** Echocardiographic results at 8 weeks after ACF operation.

Group (n)	SO (n = 20)	HF (n = 70)
LVESD (mm)	3.61± 0.31	6.21 ± 0.73**
LVEDD (mm)	6.32 ± 0.36	8.98 ± 0.59**
LVEF (%)	83.37 ± 4.21	58.93 ± 5.17**
LVFS (%)	47.19 ± 2.92	33.12 ± 5.53**
Heart rate (bpm)	410.20 ± 11.12	397.24 ± 13.50

n, the number of rats; SO, sham operation group; HF, heart failure group; LVESD, left ventricular end-systolic dimension; LVEDD, left ventricular end-diastolic dimension; LVEF, left ejection fraction; LVFS, fraction shortening. **P < 0.01 vs. SO.

**Table 2:** The parameters of cardiac function of rats in three groups after treatment.

Group (n)	SO (n = 12)	HF (n = 15)	NRG (n = 15)
Echocardiography			
LVESD (mm)	3.85 ± 0.31	6.36 ± 0.48**	4.91 ± 0.35**^#^
LVEDD (mm)	6.69 ± 1.32	9.32 ± 1.58**	7.52 ± 0.97*^#^
LVEF (%)	81.71 ± 2.01	60.81 ± 4.29**	70.24 ± 3.94*^#^
LVFS (%)	50.67 ± 5.07	31.43 ± 2.92**	41.18 ± 3.53*^#^
Hemodynamics			
LVESP (mmHg)	129.14 ± 4.82	90.79 ± 6.49**	112.04 ± 5.31*^##^
LVEDP (mmHg)	3.10 ± 0.93	12.23 ± 1.72**	7.52 ± 1.48**^##^
+dp/dt_max_ (mmHg/S)	5,678.06 ± 145.20	3,679.50 ± 134.12**	4,898.14 ± 121.04*^##^
−dp/dt_max_ (mmHg/S)	4,173.75 ± 115.51	2,457.40 ± 140.87**	3,570.37± 106.31^##^
Heart rate (bpm)	417.51 ± 21.53	424.05 ± 20.13	410.12 ± 18.12
QTc (ms)	157.13 ± 11.02	207.79± 15.91**	177.95 ± 12.12**^#^
BNP (ng/mL)	0.10 ± 0.01	0.78 ± 0.11**	0.32 ± 0.03**^##^
Heart/body weight (mg/100 g)	238.53 ± 12.47	565.04 ± 19.40**	474.23 ± 21.02**^#^
Lung/body weight (mg/100 g)	395.82 ± 20.02	598.34 ± 23.16**	503.34 ± 23.34*^#^

n, the number of rats; SO, sham operation group; HF, heart failure group; NRG, neuregulin-1β treatment group; LVESD, left ventricular end-systolic dimension; LVEDD, left ventricular end-diastolic dimension; LVEF, left ejection fraction; LVFS, fraction shortening; LVESP, left ventricular end-systolic pressure; LVEDP, left ventricular end-diastolic pressure; +dP/dt_max_, maximum velocity of ascending in intraventricular pressure; –dP/dt_max_, minimum velocity of descending in intraventricular pressure; *P < 0.05, **P < 0.01 vs. SO; ^#^P < 0.05, ^##^P < 0.01 vs. HF.

Furthermore, both heart weight/body weight ratio and the lung weight/body weight ratio were increased significantly compared with control rats (both *P* < 0.01, **Table 2**); this reflected the marked heart and pulmonary congestion. NRG-1β treatment can alleviate these changes in HF rats. These results indicated that NRG-1β significantly improved the cardiac function in rat with volume-overload HF.

### Effects of NRG-1β on L-Type Ca^2+^ Current (I_Ca-L_)

**Figure 2:**
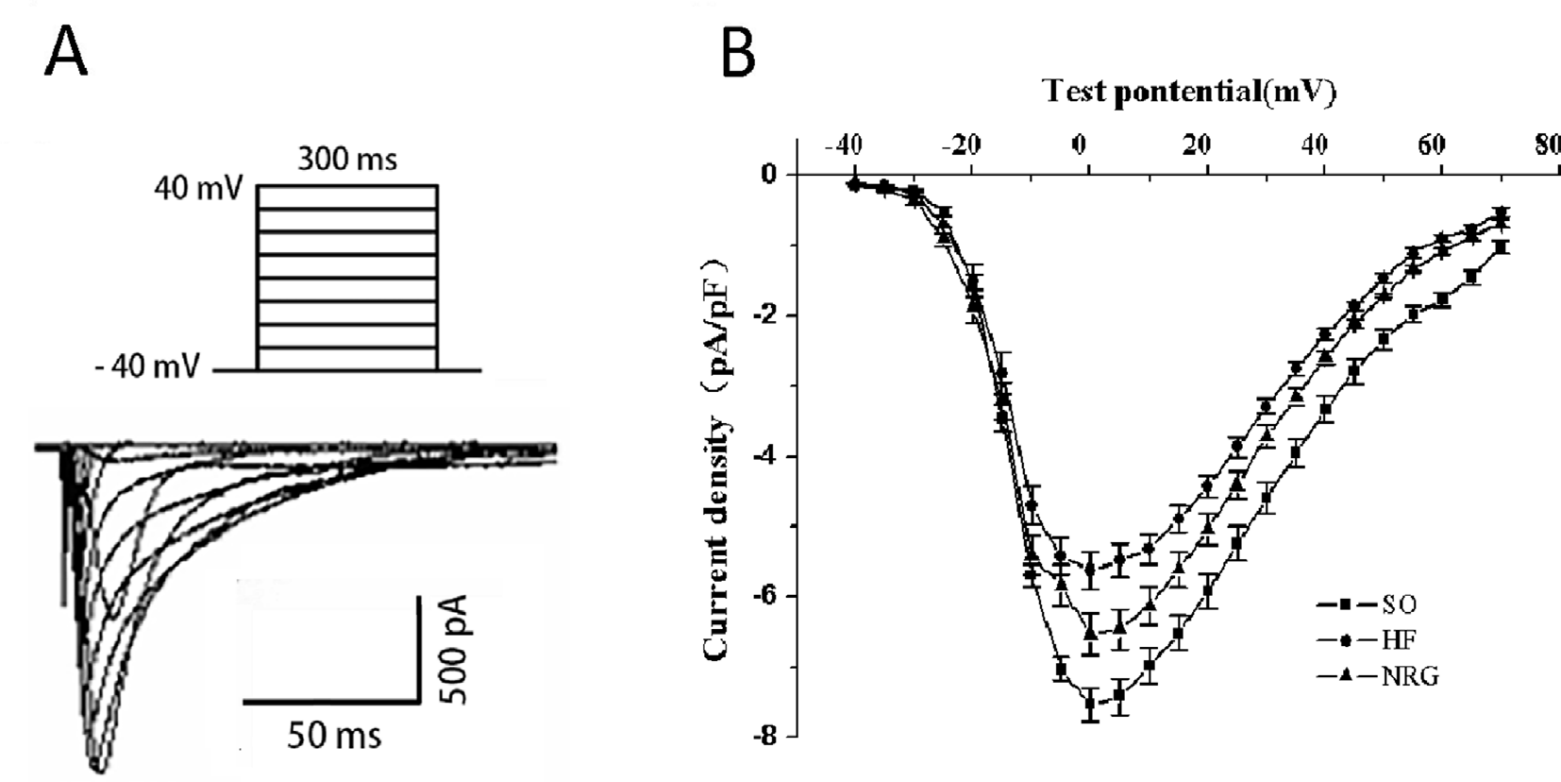
Effects of NRG-1β on I_Ca-L_ in rat ventricular myocytes. **(A)** Stimulated protocol and original current recording. **(B)** I–V relationship of I_Ca-L_. For obtaining the current-voltage (I–V) curves and steady-state activation curves, the holding potential was set at −40 mV, and I_Ca-L_ was elicited by a single pulse of 300 ms to +70 mV from the holding potential in 5-mV increments at 0.1 Hz. The current amplitude was normalized to the cell capacitance (current density, pA/pF).

Ca^2+^ handling plays pivotal roles in the normal function of cardiomyocyte; to explore the role of NRG-1β in HF, we detected the effect of on L-Type Ca^2+^ current (I_Ca-L_) in failing cardiomyocytes using whole-cell patch clamp recording. The threshold for activation of I_Ca,L_, and potential of the peak current was −40 and 0 mV, respectively (**Figure 2**). As shown in **Table 3**, the peak I_Ca-L_ density (amplitude normalized to cell membrane capacitance) in the HF group (−5.62 ± 0.15 pA/pF) was lower than that in the SO group (−7.54 ± 0.13 pA/pF) (*P* < 0.01) at the test potential of 0 mV, whereas the peak I_Ca-L_ density was markedly increased when compared with that of the HF (−6.54 ± 0.17 pA/pF *vs.* HF group, *P* < 0.05). Moreover, NRG-1β did not change the threshold (−40 mV) of activation for I_Ca-L_ or the potential (0 mV) of the peak current.

**Table 3:** I_Ca-L_ and I_Ca-L_ kinetic parameters from myocytes in different groups.

Group	N	SO	HF	NRG
I_Ca-L_/C (pA/pF)	8	−7.54 ± 0.13	−5.62 ± 0.15**	−6.54 ± 0.17**^##^
Activation V_1/2_ (mV)	9	−13.55 ± 3.65	−10.96 ± 4.82	−14.30 ± 7.70
Activation slope (k)	9	4.23 ± 0.93	5.91 ± 1.14	5.3 ± 1.67
Inactivation V_1/2_ (mV)	9	−25.04 ± 8.58	−26.04 ± 7.76	−27.31 ± 6.18
Inactivation slope (k)	9	4.21 ± 0.83	4.58 ± 0.99	5.89 ± 2.22
Fast recovery time constants (杈τ1) (ms)	6	788.78 ± 266.67	824.91 ± 238.27	800.35 ± 249.90
Slow recovery time constant (杈τ2) (ms)	6	58.80 ± 10.96	57.45 ± 10.71	65.31 ± 13.88

n, the number of cells recorded from 3 to 5 rats. *P < 0.05, **P < 0.01 vs. SO; ^#^P < 0.05, ^##^P < 0.01 vs. HF.

### Effect of NRG-1β on Steady-State Activation and Inactivation Kinetics of I_Ca-L_

**Figure 3:**
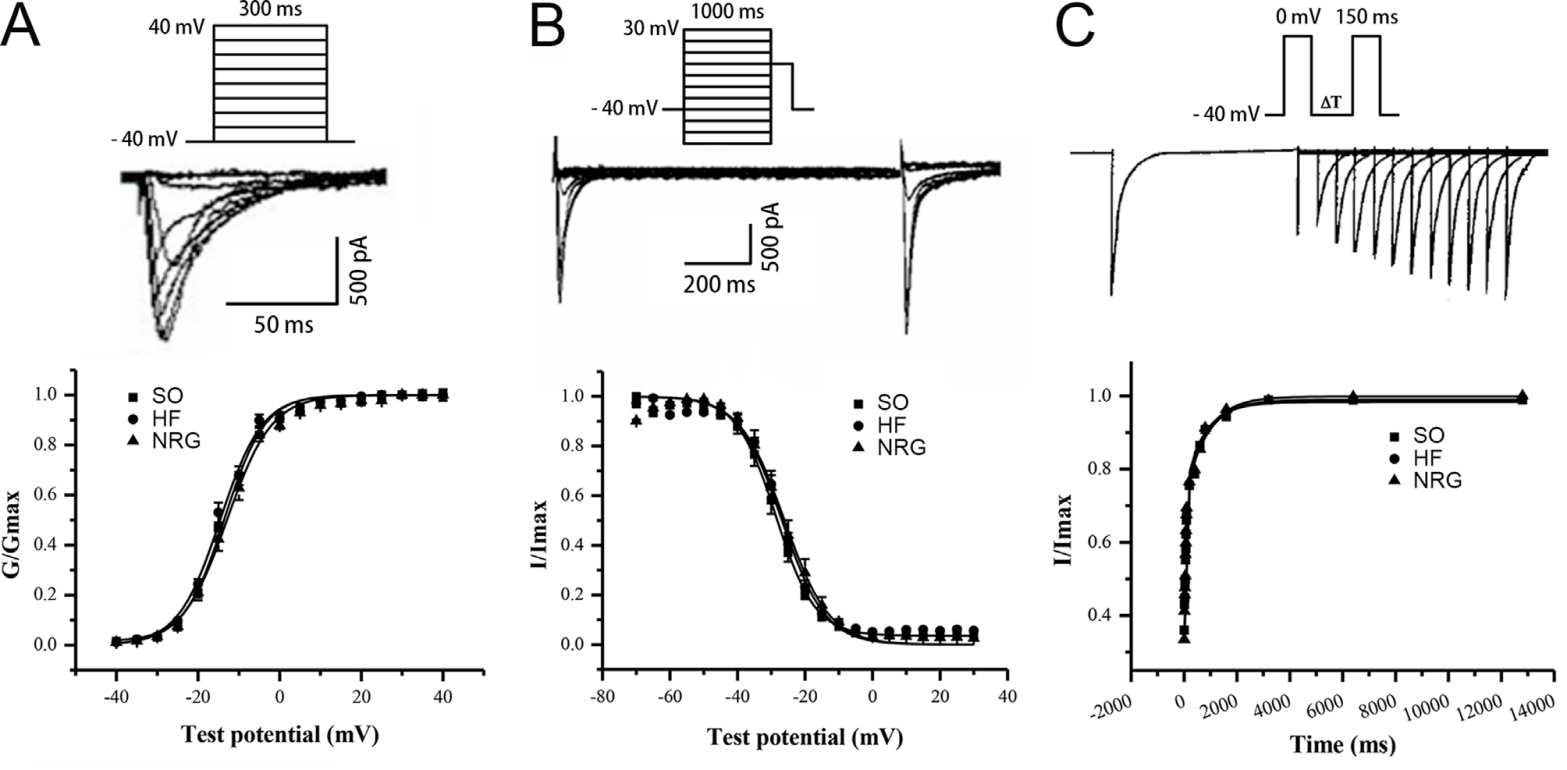
Kinetics of I_Ca-L_. **(A)** Steady-state activation curve of I_Ca-L_. **(B)** Steady-state inactivation curve. **(C)** Biexponential recovery from inactivation of I_Ca-L_. The cells were depolarized from −40 to 0 mV over 150 ms, and various interpulse (10–12,800 ms) were applied. NRG-1β did not affect the time dependence of I_Ca-L_ recovery from inactivation.

**Figure 3** showed the voltage-dependent kinetics of the steady-state activation (**Figure 3A**) and inactivation (**Figure 3B**) of I_Ca-L_, which were obtained through conventional protocols. Data on the conductance/voltage activation and inactivation curves were best fit to Boltzmann equations in the form g/g_max_ = 1/(1+exp[-(V_m_-V_1/2_)/k)] and I/I_max_ = 1/[1+exp(V_m_-V_l/2_)/k)], respectively. V_m_ is the voltage of the conditioning pulse, V_1/2_ is the potential of half activation or inactivation, and k is the slope factor. NRG-1β did not markedly influence the activation and inactivation properties of ICa-L (**Table 3**).

### Effect of NRG-1β on the Time Dependence of I_Ca-L_ Recovery from Inactivation

The time dependence of I_Ca-L_ recovery from inactivation was studied using a double-pulse protocol. Averaged data were plotted as a function of the interpulse interval, and a biexponential function fitted the data according to the equation y = A_f_[1-exp(-t/τ_f_)]+A_s_[1-exp(-t/τ_s_)], where t is the time, A_f_ and A_s_ are the amplitudes of the fast and slow recovery phase, respectively, and τ_f_ and τ_s_ are the corresponding time constants. **Figure 3C** indicates that NRG-1β did not affect the time dependence of I_Ca-L_ recovery from inactivation. Τ1 and τ2 were 788.78 ± 266.67 and 58.80 ± 10.69 ms in the SO group, 824.91 ± 238.27 and 57.45 ± 10.71 ms in the HF group, and 800.35 ± 249.90 and 65.31 ± 13.88 ms after treatment with NRG-1β (*P* > 0.05), respectively.

### Effect of NRG-1β on Calcium Handling Proteins of Rat Ventricular Myocytes

**Figure 4:**
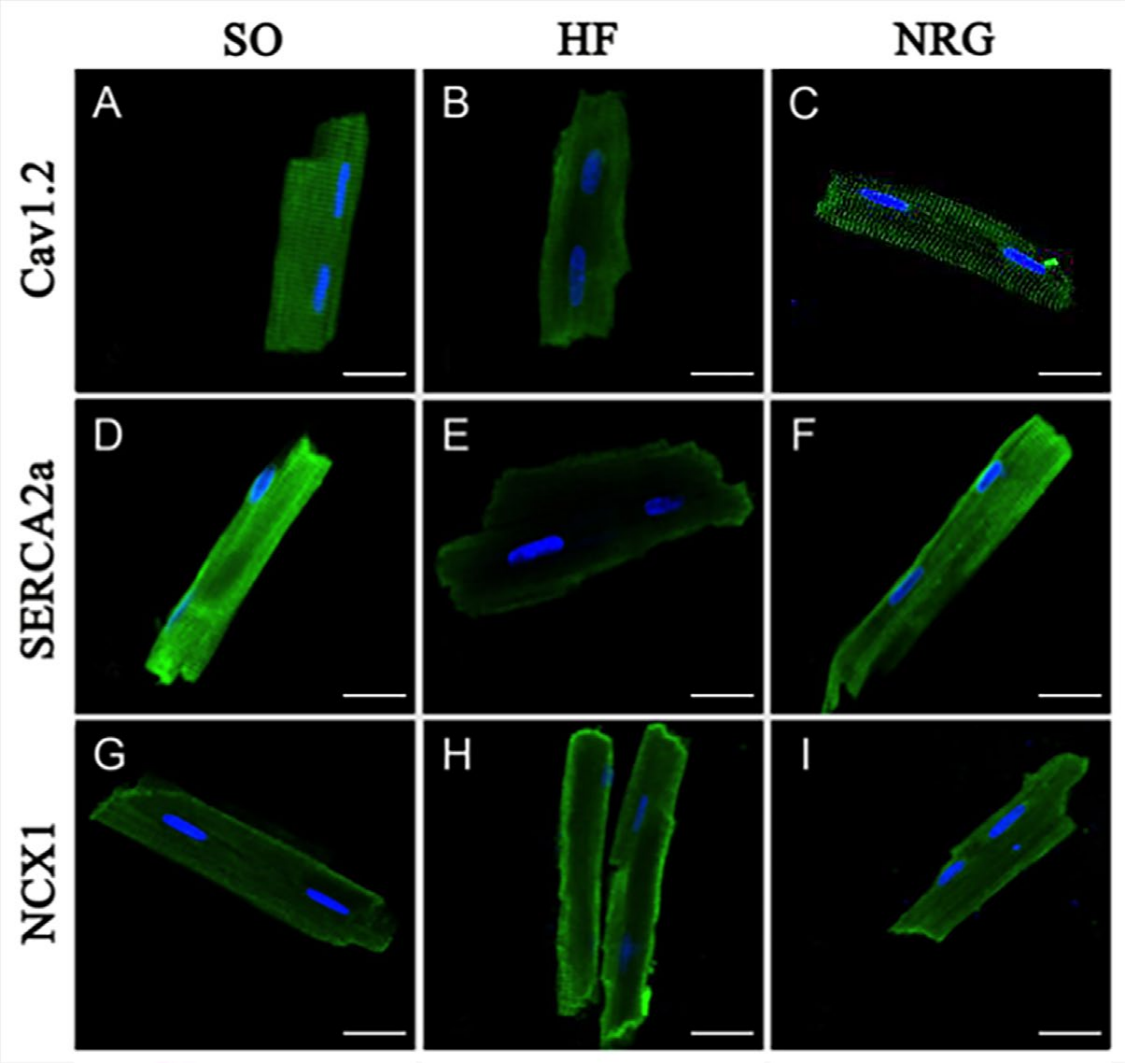
Expressions of calcium handling proteins. The confocal image showed fluorescein isothiocyanate (green)-labeled Cav1.2, SERCA2a, and NCX1 expressed in isolated rat cardiac ventricular myocytes in SO (left panel), HF (middle panel), and NRG group (right panel). Nuclei were stained with DAPI (blue). The scale bar represents 20 μm (n = 5 for each set of staining).

Immunocytochemistry and confocal microscopy were used to detect the expression of Ca^2+^ handling proteins in isolated adult heart cells. **Figure 4** showed the rat ventricular myocytes that were labeled using specific antibodies directly against Cav1.2, SERCA2a, and NCX1 in the SO (left panel), HF (middle panel), and NRG groups (right panel).

**Figure 5:**
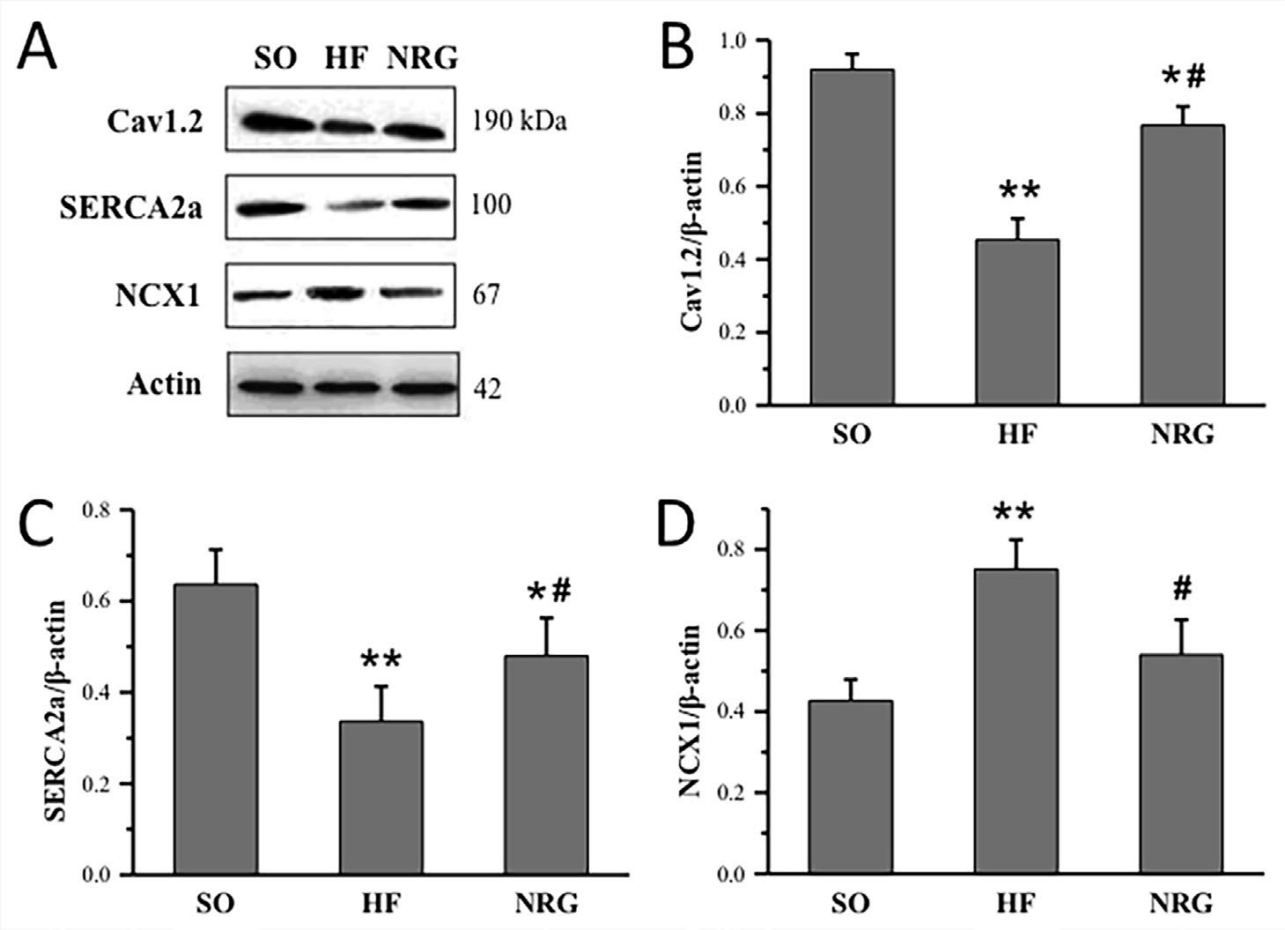
Quantitative analysis of calcium handling proteins expressions. **(A)** Representative expression of Cav1.2, SERCA2a, and NCX1, β-actin as loading controls. **(B)** Comparison of Cav1.2 protein expression (n = 3 rats). **(C)** Comparison of SERCA2a protein expression (n = 4 rats). **(D)** Comparison of NCX1 protein expression (n = 4 rats). *P < 0.05, **P < 0.01 *vs.* SO; ^#^ *P* < 0.05, ^##^ *P* < 0.01 *vs.* HF.

Western blot analysis was conducted in three groups to determine whether NRG-1β regulated protein expression levels of Cav1.2, SERCA2a, and NCX1 in the left ventricular myocardium. As shown in **Figure 5**, the expression levels of Cav1.2 and SERCA2a in the HF group were significantly downregulated compared with that in the SO group (*P* < 0.01), whereas NCX1 was upregulated in the HF group. After treatment with NRG-1β, Cav1.2 and SERCA2a protein expressions in the NRG group were significantly increased (both *P* < 0.05 *vs*. HF), and NCX1 protein expression was significantly decreased (*P* < 0.05 *vs*. HF).

## Discussion

This study demonstrated that 1) NRG-1β improves cardiac function of rats with volume-overload HF, 2) increases the Ca^2+^ current density of LV cardiomyocytes, and 3) promotes the expression levels of Ca^2+^ handling proteins, including Cav1.2, SERCA2a, and NCX1, in the LV tissue.

### NRG-1β in Treatment of Volume-Overload HF

Recent studies have revealed that recombinant NRG-1β improves cardiac contractility and relaxation in chronic HF rat models induced by ischemic, dilated, and viral cardiomyopathy (Liu et al., 2006). Similar improvements in cardiac function have also been observed in dogs (Liu et al., 2006) and primates (Li et al., 2007). A series of early studies involving recombinant NRG in humans have been undertaken in several countries, and the preliminary results have shown that NRG-1β improves cardiac structure and function in HF patients (Gao et al., 2010; Jabbour et al., 2011). Nonetheless, the exact mechanism underlying NRG-1β’s mode of action has not been established.

### Downregulated L-type Ca^2+^ Channel Protein in Volume-Overload HF

The fast cycling of Ca^2+^ between internal stores and myofilaments is essential for cardiac contraction and relaxation. Emerging evidence indicated that in HF, impaired intracellular Ca^2+^ handling leads to abnormal ECC and contraction. I_Ca-L_ is a prerequisite for initiating and maintaining intracellular Ca^2+^ handling (Seisenberger et al., 2000). Changes of I_Ca-L_ are controversial in diverse HF models, including decreased (Mukherjee et al., 1998) or unchanged (He et al., 2001), which highlight the complexity of HF. The variations may be a result of different animal models of HF at the various stages of HF. Mukherjee et al. (1998) reported that the I_Ca-L_ density was reduced by over 25% after 3 weeks of pacing with no marked change in the kinetic parameter, which is consistent with our findings. Our data demonstrated that I_Ca-L_ density decreased significantly in the LV myocardial cells in HF rats. However, the dynamic characteristics of I_Ca-L_ were not affected. These results indicate that the amount of functional Ca^2+^ channels might be affected.

### Reduced I_Ca-L_ Density is Closely Related to Decreased Protein Expression of the Ca^2+^ Channel

The previous study demonstrated that severe decreases in cardiac protein expression of the α1C subunit in α1C^−/+^ mice were correlated with a corresponding reduction in I_Ca-L_, which led to an aggravation of cardiac hypertrophy and deterioration of cardiac function (Goonasekera et al., 2012). Hu et al. (2011) reported that I_Ca-L_ density decreased by approximately 50% because of downregulation of Cav1.2 expression at the mRNA and protein level in rat HF. The present study demonstrated that the downregulation of Cav1.2 expression contributed to the I_Ca-L_ density decreased, which was partially eliminated by NRG-1β treatment. Furthermore, the acute effect of NRG (1 µg/ml) on ventricular cardiomyocytes isolated from sham-operated rats and HF rats showed that NRG significantly increases I_Ca-L_ from HF rats, but not from SO rats (**Supplemental Figure III**). These data suggest NRG directly increases I_Ca-L_.

The present study demonstrated that chronic treatment with NRG-1β significantly increased I_Ca-L_ density with no change in kinetic parameters. However, we cannot verify whether I_Ca-L_ underlies NRG’s effect on HF rats or just secondary to the improvement of HF rats. These data suggest NRG also directly increases I_Ca-L_. Activation of the sympathetic nervous system and the RAAS system in HF produces alterations in neurohumoral modulation and influences the functions, trafficking, and membrane targeting of L-type Ca^2+^ channel (Takahashi et al., 1992; Hu et al., 2011). Lipsky et al. (2008) found that persistent activation of β-adrenergic receptors induced dysfunction of L-type Ca^2+^ channels *via* internalization of cardiac Cav1.2 channel complexes. Moreover, the β-adrenergic receptor signaling pathway is abnormally regulated. L-type Ca^2+^ channels can be modulated through activation of β-adrenergic receptors (β-Ars), which leads to an increase in I_Ca-L_ density as a result of phosphorylation by cAMP-dependent protein kinase A. Sustained β-AR activation in HF induces desensitization of β-Ars and leads to a reduction of I_Ca-L_ density.

### NRG-1β Facilities Ca^2+^ Handling in Volume-Overload HF

Moreover, I_Ca-L_ is tightly regulated by calcium handling protein such as Cav1.2, SERCA2a, or NCX1; calcium and calcium handling play pivotal roles in normal cardiac function. Therefore, we investigated whether NRG was improving cardiac function of HF rats *via* a reversal of disrupted Ca^2+^ handling proteins. Our Western blot analysis indicated that NRG-1β partially increased the expression level of SERCA2a and reduced the NCX1 in HF rats.

Two pathways are responsible for diastolic Ca^2+^ removal. SERCA2a is a Ca^2+^ ATPase that transfers Ca^2+^ from the cytosol of the cell to the lumen of the SR during cardiac muscle relaxation. NCX is an antiporter membrane protein that removes Ca^2+^ from cells. The function and change in the activity of these two proteins directly influence cardiac systolic and diastolic function. Previous reports have suggested that depressed SERCA2a function and enhanced NCX activity lead to reductions in the force of contraction and an increase in diastolic tension during HF (O’rourke et al., 1999). Our data showed that decreased SERCA2a and increased NCX protein expression from the failing myocardium were consistent with findings from other studies. Moreover, these changes can be attenuated by NRG-1β treatment.

### Limitations

This initial study investigated the cardioprotective effect of NRG-1β in HF model, which might relate to the expression level of calcium handling associated proteins. However, the direct impact on the function of calcium handling was missing. Further studies are warranted to identify the functional protection in calcium handling, as well as the elimination of protection by blockage of the key protein. Also, the present study did not distinguish the T-type Ca2+ current out from the L-type with specific blocker. However, the L type Ca2+ current was recorded with specific pipette solution, and bath solution combines with testing protocol, which gave typical L-type Ca2+ current kinetics.

## Conclusion

The present study suggested that NRG-1β exerts cardioprotective effects in volume-overload HF rats. The underlying mechanism might be that NRG-1β prevented the loss of L-type Ca^2+^ channels function and expression, as well as modulated the expression level of SERCA2a and NCX1.

## Author Contributions

XW and AM contributed to the conception and design of the study. XW and XZ contributed to the acquisition of data. XW, HL, and FL performed the analysis and interpretation of data. XW and JG drafted the article. All authors approved the final version for submission.

## Funding

This work was supported by Science and Technology Program for Public Wellbeing (2012GS610101 to AM) and the Foundation and Frontier Technology Research Project in Henan province (No. 142300410191 to XW) and the Henan province science and technology project (No. 182102310182 to XW). 

## Conflict of Interest Statement

The authors declare that the research was conducted in the absence of any commercial or financial relationships that could be construed as a potential conflict of interest.

## Abbreviations

Cav1.2, L type voltage-dependent Ca^2+^ channel alpha 1C subunit; ECC, Excitation-contraction coupling; HF, Heart failure; NCX, Sodium-calcium exchanger (NCX); NRG-1β, Neuregulin-1β; SERCA2a, SR Ca^2+^ ATPase; SO, Sham-operated
