# Supplementary material for: Neuregulin-1β Partially Improves Cardiac Function in Volume-Overload Heart Failure Through Regulation of Abnormal Calcium Handling
Source: Front Pharmacol. 2019 Jun 21;10:616. doi: 10.3389/fphar.2019.00616 (PMC6597678; doi:10.3389/fphar.2019.00616)

**Neuregulin-1β partially improves cardiac function in volume-overload heart failure through regulation of abnormal calcium handling**

Xuehui Wang, PhD^1#^, Xiaozhen Zhuo, PhD^2^, Jie Gao, MSc^2^, Huibing Liu, MSc^1^, Fei Lin, PhD^1^, Aiqun Ma, MD, PhD^2#^

**Supplemental Materials**

**Figure I** The representative echocardiography image and ECG traces

1. The representative echocardiography images


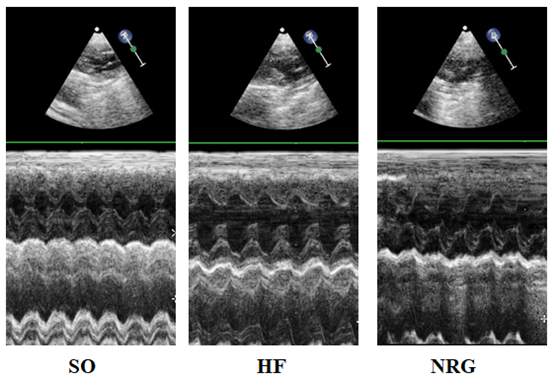


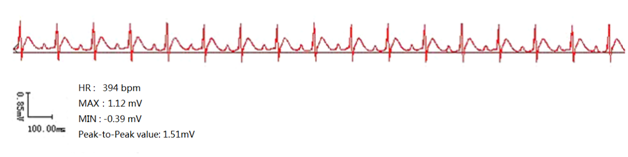
B. The representative ECG traces

**Figure II**  Negative control of immunofluorescence with Alexa Fluor 488-conjugated goat anti-rabbit IgG (H+L).


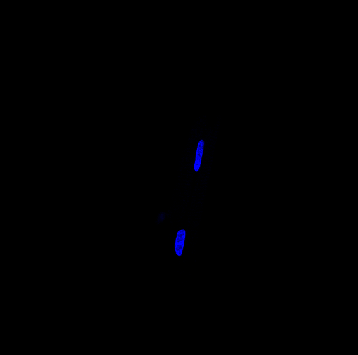


**Figure III** The acute effect of NRG (1 µg/mL) on I_Ca-L_ of ventricular cardiomyocytes isolated from (A) sham-operated rats and (B) HF rats.

1.
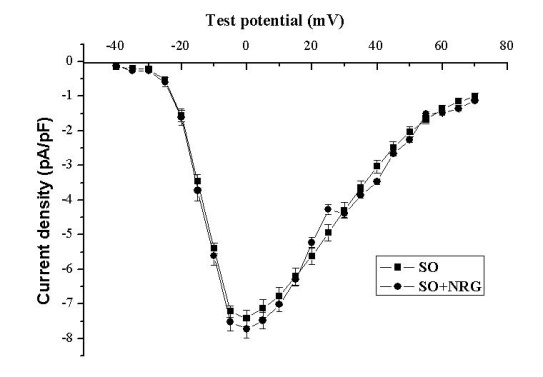


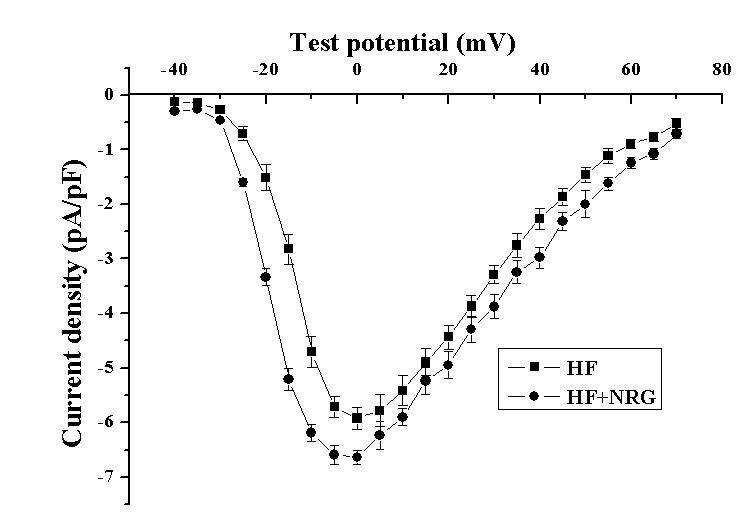

Supplement: Supplementary file 1 [file DataSheet_1.docx]
